# Correlates of physical activity and sedentary time in young adults: the Western Australian Pregnancy Cohort (Raine) Study

**DOI:** 10.1186/s12889-018-5705-1

**Published:** 2018-07-25

**Authors:** Erin K. Howie, Joanne A. McVeigh, Elisabeth A. H. Winkler, Genevieve N. Healy, Romola S. Bucks, Peter R. Eastwood, Leon M. Straker

**Affiliations:** 1 Department of Health, Human Performance and Recreation, University of Arkansas, Fayetteville, AR USA; 2 School of Physiotherapy and Exercise Science, Curtin University, Perth, Australia; 3 School of Occupational Therapy and Social Work, Curtin University, Perth, Australia; 4 School of Physiology, University of the Witwatersrand, Johannesburg, South Africa Exercise Laboratory, School of Physiology, University of Witwatersrand, Johannesburg, South Africa; 5 School of Public Health, The University of Queensland, Brisbane, Australia; 6 Baker Heart & Diabetes Institute, Melbourne, VIC Australia; 7 School of Psychological Science, University of Western Australia, Perth, Australia; 8 Centre for Sleep Science, School of Human Sciences, University of Western Australia, Perth, Australia

**Keywords:** Accelerometer, Epidemiology, Life-course

## Abstract

**Background:**

The socioecological model proposes a wide array of factors that influence behaviours. There is a need to understand salient correlates of these activity behaviours in a specific population. However, few studies identified socio-demographic, behavioural, physical, and psychological correlates of objectively-assessed physical activity and sedentary time in young adults.

**Methods:**

This was a cross-sectional analysis of participants in the Raine Study (a pregnancy cohort started in 1989)**.** Australian young adults (mean 22.1 years ± SD 0.6) wore Actigraph GT3X+ accelerometers on the hip 24 h/day for seven days to assess moderate-to-vigorous physical activity (MVPA) and sedentary time (*n* = 256 women, *n* = 219 men). Potential correlates were assessed via clinical assessment and questionnaire and included socio-demographic variables (ethnicity, relationship status, work/study status, education, mothers education), health behaviours (food intake, alcohol consumption, smoking status, sleep quality), and physical and psychological health aspects (anthropometrics, diagnosed disorders, mental health, cognitive performance). Backwards elimination (*p* < 0.2 for retention) with mixed model regressions were used and the gender-stratified analyses were adjusted for demographic variables, waking wear time and number of valid days.

**Results:**

Increased time spent in MVPA was associated with: being single (IRR 1.44 vs in a relationship living together, 95%CI: 1.17, 1.77, *p* = .001) in women; and better sleep quality in men (lower scores better IRR 0.97, 95%CI: 0.93, 1.00). Less time spent sedentary was associated with: lower mother’s education (− 32.1 min/day, 95%CI -52.9, 11.3, *p* = 0.002 for having mother with no university degree vs at least a baccalaureate degree) and smoking (− 44.3 min/day, 95%CI: - 72.8, − 15.9, *p* = .0002) for women; lower education status (− 32.1 min/day, 95%CI: -59.5, − 4.8, *p* = 0.021 for having no university degree vs at least a baccalaureate degree) and lower depression scores in men (− 2.0, 95%CI: - 3.5, − 0.4, *p* = 0.014); more alcoholic drinks per week for women (− 1.9 min/day, 95%CI: -3.1, − 0.6, *p* = 0.003) and men (− 1.0, 95%CI: -1.8, − 0.3, *p* = 0.007).

**Conclusions:**

Less desirable correlates were associated with positive levels of activity in young Australian adult women and men. Interventions to increase MVPA and decrease sedentary activity in young adults need to specifically consider the life stage of young adults.

**Electronic supplementary material:**

The online version of this article (10.1186/s12889-018-5705-1) contains supplementary material, which is available to authorized users.

## Background

Increasing physical activity levels, and decreasing time spent sedentary are key public health goals [1]. There are several known correlates of physical activity and sedentary behaviours in adult [2, 3] and child populations [4, 5]. These activity correlates exist at multiple levels in an socioecological framework and include sociodemographic factors of education level, income, and marital status [3, 4, 6] physical factors such as body composition and health conditions, psychological factors including mental health [7] and behavioural factors such as diet and smoking [4].

Identifying and understanding these sociodemographic, health and behavioural correlates in specific populations is a first step in designing optimal interventions [2, 8]. Firstly, identifying sociodemographic factors associated with physical activity behaviours will help to design targeted interventions for populations in need. Examples of interventions specifically designed for adults with a low-income have been reviewed and found to be successful [9]. Secondly, understanding health factors associated with activity behaviours may identify special considerations for safely and optimally promoting physical activity in specific populations. For example, in individuals with metabolic syndrome, interventions need to target both physical activity and sedentary behaviours [10] and adults with both depression and cardiovascular disease may be in higher need of physical activity interventions [11]. The combination of physical activity behaviours and health factors may also highlight unique interactions as joint targets of interventions, such as the interaction between obesity with physical activity and sedentary behaviour [12]. Lastly, an understanding of how multiple health behaviours coexist will help create effective and efficient interventions to positively affect health. For example, to achieve desired health benefits, such as improved mental health, both physical activity and sleep may need to be addressed simultaneously in an intervention [13]. Physical activity and sleep likely have a complex relationship where regular physical activity may improve sleep, insufficient sleep may decrease physical activity, and extreme physical activity may harm sleep [14]. While the socioecological model proposes a wide array of factors that influence behaviours, understanding the salient correlates of these activity behaviours in a specific population is an essential step to informing prospective longitudinal studies by focusing in on potential predictors of behaviour and ultimately targets for interventions.

Correlates of physical activity and sedentary behaviour may vary by life stages [2–5]. For example, children’s physical activity levels were associated with parent physical activity and parent support, whereas adolescents’ physical activity levels were associated with perceived competence, attitude, perceived behavioural control [2]. Only a limited number of studies have specifically examined correlates of physical activity and sedentary behaviours in young adults. Young adults are a unique population undergoing several life transitions [6] which may influence their decreasing activity levels [15, 16]. Life transitions provide an opportunity, but also a risk of changing physical activity habits. In younger age groups, adolescents have reported transitioning away from structured sport as school demands increased [17]. Young adults undergo further life transitions as they move away from home, participate in university or employment, and begin their own families. A clear understanding of physical, psychological, behavioural and interpersonal correlates of physical activity and sedentary behaviours at this critical transition period is essential to design interventions to help maintain or increase physical activity levels into adulthood.

This study used data from the Western Australian Pregnancy Cohort (Raine) Study to examine a wide range of potential correlates of daily moderate-to-vigorous physical activity (MVPA) and sedentary time in young adults. This study extends previous research investigating correlates of activity and sedentary time in that it used an objective measure of activity (i.e. accelerometry) in young adults rather than self-report measures, the latter of which are prone to bias and poor validity. [18] Given the richness of Raine Study data, we were able to examine a variety of potential correlates including sociodemographic, behavioural, physical and psychological factors.

## Methods

Study Design and Participants. This study is part of a larger pregnancy cohort: the Raine Study. Detailed methods of the 22 year old data collection [19], activity findings [20], and representativeness of the study sample [21] have been previously published. In brief, expecting mothers were recruited between 1989 and 1991 from which 2868 children entered the study. The current study includes data from the 22 year follow-up (data collected between 2012 and 2014) and is supplemented by earlier demographic information. Written, informed consent was obtained from the mother until the children reached the age of 18 and provided their own consent. Follow-ups of the study families were approved by the respective ethics committees at King Edward Memorial Hospital, Princess Margaret Hospital for Children, the University of Western Australia, and/or Curtin University.

### Measures

At the 22 year follow-up, participants attended the Centre for Sleep Science at the University of Western Australia where they completed selected tests from the Cogstate battery, a sleep quality scale, a food frequency evaluation, a clinical assessment and were fitted with an activity monitor. They also returned a written questionnaire (available at rainestudy.org.au) which they were asked to complete prior to the clinic visit. A description of the variables used in the analyses can be found in Additional file 1: Table S1.

Potential correlates of physical activity and sedentary time were selected, a priori*,* based on literature and public health theory grouped into domains of sociodemographic, health behaviours, and physical & psychological health variables.

Sociodemographic Domain. Data on education (at least a bachelor’s degree vs not), working and studying status (working full time, studying full time, studying or working part-time, not working or studying) and relationship status (single, in a relationship but not living together, in a relationship or married and living together) were collected via the questionnaire. Ethnicity and parent education levels were reported by parents at the age 8 assessment.

#### Behavioural domain

Daily servings of fruit and vegetables, and the total energy consumed in kilojoules (adjusted for total body weight), were derived from the Dietary Questionnaire for Epidemiological Studies Version 2 from the Cancer Council (Victoria, Australia) Food Frequency questionnaire. Sleep quality (past month) was assessed via the Pittsburgh Sleep Quality Index (PQSI). Scores ranged from 0 to 16 with higher scores representing poorer sleep quality [22]. The number of standard alcoholic drinks per week, and current smoking status (yes or no to currently smoking cigarettes/cigars) were measured in the general questionnaire.

Physical and psychological health domain.The physical component scale of the Short Form-12 Health Survey version 2 (SF-12) [23], which has been cross-validated in Australian populations [24], was used to capture perceived physical health, with higher scores indicating better health. The summary subscale scores of the Depression Anxiety Stress Scales (DASS-21) [25] were calculated, with higher scores (out of 42) indicating poorer mental health.

The Cogstate (Cogstate Ltd., Melbourne, Australia) battery is a computerised battery of cognitive tests. [19] For these analyses, the card detection (speed of processing), card identification (vigilance and visual attention) and one back (attention and working memory) tasks were used [26]. Participants were presented with instructions on screen and self-administered the test. Data were processed and reaction times are presented as log_10_-transformed values.

Height, weight, and waist circumference were measured by research staff during the clinical assessment according to a standardized protocol [19]. Additionally, the number of current diagnosed disorders (e.g. asthma, diabetes, eating disorders) was asked on the written questionnaire.

#### Physical activity and sedentary time

Participants were instructed to wear an activity monitor (Actigraph GT3X+, Pensacola, FL) on their right hip for 24 h/day for the following seven days except during water-based activities. Raw data were collected at a frequency of 30 Hz, with data for this analyses processed in 60 s epochs. Data were processed using a custom automated algorithm [27] to identify waking wear time in SAS (version 9.3, SAS Institute, Cary, NC, USA). Age-appropriate cutpoints were used to determine, from the vertical axis, the number of daily minutes in MVPA (≥1952 counts per minute) [28] and time spent sedentary (< 100 counts per minute) [29]. Light intensity was not examined as it is known to have a strong inverse relationship with sedentary time [30]. A day was considered valid if it had at least 10 h of wear time. Participants with at least one valid day (*n* = 774) were included in the analyses, with sensitivity analyses conducted for participants with at least three days of valid wear.

### Analysis

To keep a consistent sample size in the backwards elimination process, only participants with valid data for all variables were included (*n* = 475). Patterns of missing data were explored and the included sample was compared to the excluded sample using t-tests, Mann-Whitney U, or chi-squared tests. Significance was set at *p* < 0.05, two-tailed.

Separate analyses were conducted for the two outcomes of physical activity (MVPA min/day) and sedentary time (min/day). Models were stratified a priori by sex due to known gender differences in physical activity and their differential associations with health found in this cohort [31] and others [32, 33] as well as differential physical activity intervention effects in men and women [34]. To examine the relationships of potential correlates with MVPA and sedentary time, mixed generalized linear models, with days clustered within individuals (based on a hierarchical linear modelling approach) were used. A negative binomial distribution with log link was used for MVPA models and a normal Gaussian distribution was used for sedentary time; relative rates (RR) and beta coefficients are presented, respectively. All models were adjusted for wear time during waking hours and the number of valid days to limit any effects resulting from more time or days of wear. Residual plots were checked for distribution of errors and model fit. Variance Inflation Factors were examined for all models and no collinearity was found (Variance Inflation Factor < 2.5).

A sequential stepped approach was used to fit models. Bivariate, or unadjusted, analyses are included in Additional file 1: Table S2 to enable comparisons to other studies. This stepped approach of adjusting was selected to adjust for potential confounders but not to adjust for related causes (variables likely to be on the same causal pathway) [35]. As sociodemographic variables are most likely to be confounders, all models were adjusted for the significant sociodemographic variables as selected below. Variable selection was by backwards elimination with *p* < 0.2 for retention. Variables that were eliminated were added back one-by-one to the model to verify exclusion.

To examine sociodemographic factors associated with MVPA and sedentary time, potential sociodemographic correlates were examined individually, and then adjusted for each other, eliminating variables with *p* > 0.2. To examine which behaviours co-occurred with MVPA and sedentary time, potential behavioural correlates were examined individually adjusted for sociodemographic confounders, and then adjusted for each other. To examine which aspects of physical and psychological health were associated with MVPA and sedentary time, potential health correlates were examined individually adjusted for sociodemographic confounders, and then other health behaviours. We did not mutually adjust the health factors to examine independent relationships as health factors are known to commonly co-occur, thus identifying which independent health factors are most related was not of interest.

## Results

A total of 1239 participants had at least one variable included from the assessment at age 22. Of those, 475 (*n* = 256 women, *n* = 219 men) had valid accelerometer data and all 19 potential correlate variables and were included in the analyses. Out of the 774 with accelerometry, 92 were missing 1 of the potential correlates, 19 were missing 2, 83 were missing 3 variables and 105 were missing 4 or more potential correlates. A description of the sample included in the analyses can be found in Table 1 and compared to participants with available data in the Additional file 1: Table S3. Generally, the included sample was of slightly higher socioeconomic status than the Raine participants as a whole.

**Table 1:** Activity, socio-demographic, behavioural, and physical and psychological health of the analytic sample, mean (SD), median (25th,75th), or n (%)

Domain	Variable	Women (*n* = 256)	Men (*n* = 219)
Accelerometer Variables	MVPA (average min/ day)	27 (16.2, 41.3)	34.1 (20.3, 52.1)
	Sedentary (average min/day)	569.7 (85.1)	549.1 (92.8)
	Wear time (average min/day)	903.3 (90.7)	905.3 (89.3)
	Number of valid days	5.5 (2.3)	5.6 (2.3)
Sociodemographic	Ethnicity (% Caucasian mother and father)	218 (85.2%)	186 (84.9%)
	Mother’s education (university vs no university)	70 (27.3%)	65 (29.7%)
	Education (university vs no university)	89 (34.8%)	52 (23.7%)
	Studying/Working (vs neither)		
	*Part-time*	42 (16.4%)	31 (14.2%)
	*Full time studying*	112 (43.8%)	67 (30.6%)
	*Full time working*	81 (31.6%)	89 (40.6%)
	Relationship (vs single)		
	*Relationship not living together*	90 (35.2%)	76 (34.7%)
	*Relationship living together or married*	54 (21.1%)	36 (16.4%)
Behavioural	Diet		
	*Fruit & Vegetable (serves/day)*	7 (1, 8)	6 (1, 8)
	*Total energy (kJ/kg/day)*	89.0 (66.5, 116.6)	112.2 (83.6, 150.2)
	Alcohol (drinks/wk)	3.1 (0.6, 7.5)	6 (1.3, 14.5)
	Smoking	31 (12.1%)	35 (16.0%)
	Sleep quality (0–16, higher scores indicate poorer sleep)	4 (3, 6)	4 (3, 6)
Physical & Psychological Health	Waist Circumference (cm)	80.0 (14.0)	85.2 (11.2)
	# Diagnosed disorders (current)	2 (1, 3)	1 (0, 2)
	SF-12 physical component	53.5 (6.6)	54.8 (4.8)
	DASS-21 (score range 0 to 42, higher scores indicate poorer mental health)		
	*Depression*	4 (2, 12)	2 (0, 8)
	*Anxiety*	4 (0, 8)	2 (0, 6)
	*Stress*	10 (4, 16)	6 (2, 10)
	Vigilance (Cogstate – Identification, log10 reaction time in seconds)	2.6 (2.6, 2.7)	2.6 (2.6, 2.7)
	Speed of processing (Cogstate – Detection, log10 reaction time in seconds)	2.4 (2.4, 2.5)	2.4 (2.4, 2.5)
	Attention & working memory (Cogstate – One Back, log10 reaction time in seconds)	2.8 (2.8, 2.9)	2.8 (2.7, 2.8)

### MVPA

The findings for MVPA can be found in Table 2. In the sociodemographic domain, for young women, more time spent in MVPA was statistically and independently associated with a single relationship status (compared to living with a partner or married). For young men, none of the sociodemographic variables were statistically significant at *p* < 0.05; the only sociodemographic variable retained in the model was mother’s education (*p* = 0.051), where having a mother with at least a university degree was associated with more time spent in MVPA.

**Table 2:** Correlates of MVPA (unstandardized rate ratios, 95%CI, *p*-value)

	Women		Men		Women		Men	
	Model 1: adjusted for multiple sociodemographic variables				Model 2: adjusted for sociodemographics and co-occurring behaviours			
Sociodemographic domain								
Ethnicity (vs not Caucasian)	1.22 (0.98, 1.52)	0.077	*Not included*					
Mother’s education (vs no university)	*Not included*		1.20 (0.98, 1.45)	0.075				
Education (vs no university)	*Not included*		*Not included*					
Work/Study Status (vs neither)	*Not included*		*Not included*					
*Part-time*								
*Full time studying*								
*Full time working*								
Relationship (vs single)		**0.003**	*Not included*					
*Relationship not living together*	0.87 (0.73, 1.04)	0.133						
*Relationship living together or married*	0.70 (0.57, 0.86)	0.001						
Behavioural Domain								
Fruit & Veg (serves/day)	1.01 (0.99, 1.03)	0.437	1.00 (0.97, 1.03)	0.982	*Not included*		*Not included*	
Energy (kj/kg/day)	1.00 (1.00, 1.00)	0.448	1.00 (1.00, 1.00)	0.158	*Not included*		1.001 (1.00, 1.002)	0.132
Alcohol (drinks/week)	1.01 (1.00, 1.02)	0.165	1.00 (1.00, 1.00)	0.395	1.01 (1.00, 1.02)	0.128	*Not included*	
Smoking (vs non-smoking)	1.13 (0.88, 1.45)	0.349	0.94 (0.73, 1.21)	0.636	*Not included*		*Not included*	
Sleep Quality (PQSI) (0–16 scale)	0.98 (0.94, 1.01)	0.180	0.97 (0.93, 1.00)	0.058	0.97 (0.94, 1.01)	0.139	**0.97 (0.93, 1.00)**	**0.049**
Physical & Psychological Health Domain								
Waist Circumference (cm)	0.996 (.991, 1.002)	0.196	1.00 (1.00, 1.01)	0.453	1.00 (0.999, 1.00)	0.241	1.01 (1.00, 1.02)	0.108
Diagnosed disorders (#)	0.97 (0.93, 1.00)	0.086	0.97 (0.92, 1.02)	0.193	0.98 (0.94, 1.02)	0.306	0.98 (0.93, 1.04)	0.673
Physical Health (SF12) (0–100 scale)	1.01 (1.00 (1.02)	0.183	1.00 (0.98, 1.02)	0.928	1.01 (0.99, 1.02)	0.344	0.99 (0.98, 1.02)	0.969
DASS depression (0–42 scale)	0.99 (0.98, 1.00)	0.025	1.00 (0.99, 1.01)	0.830	0.99 (0.98, 1.00)	0.078	1.01 (0.99, 1.02)	0.300
DASS anxiety (0–42 scale)	1.00 (0.98, 1.01)	0.590	0.99 (0.97, 1.01)	0.231	1.00 (0.98, 1.01)	0.767	1.00 (0.98, 1.44)	0.849
DASS stress (0–42 scale)	1.00 (0.99, 1.01)	0.559	0.99 (0.98, 1.01)	0.307	1.00 (0.99, 1.01)	0.981	1.00 (0.99, 1.02)	0.947
Vigilance (Cogstate – Identification, log10 reaction time (s))	1.26 (0.40, 4.00)	0.690	1.13 (0.27, 4.75)	0.864	1.28 (0.41, 4.02)	0.673	1.19 (0.29, 4.89)	0.811
Speed of processing (Cogstate – Detection, log10 reaction time (s))	1.27 (0.56, 2.87)	0.570	0.78 (0.25, 2.44)	0.672	1.27 (0.56, 2.88)	0.560	0.75 (0.24, 2.31)	0.618
Attention & working memory (Cogstate – One Back, log10 reaction time (s))	1.05 (0.43, 2.59)	0.910	2.38 (0.89, 6.38)	0.085	1.09 (0.45, 2.68)	0.844	2.22 (0.84, 5.9)	0.109

Model 1 adjusted for- Women: ethnicity, relationship status; Men: mother’s education

Model 2 adjusted for: Women - ethnicity, relationship status, alcohol, sleep; Men - mother’s education, energy, sleep

Bold indicates a statistically significant correlate at p<.05

In the behavioural domain for young women, no significant behavioural correlates of MVPA were identified. For young men, lower sleep quality scores (indicating better sleep) were associated with higher MVPA when adjusted for sociodemographic variables and other health behaviours; (see Model 2 in Table 2).

In the physical & psychological health domain, no physical or psychological health variables were identified as statistically significant correlates in either young women or young men in either models.

### Sedentary time

The findings for sedentary time are seen in Table 3. In the sociodemographic domain, for young women, mother’s education (having no university degree vs at least a baccalaureate degree) was the only statistically significant independent sociodemographic correlate of decreased sedentary time identified. For young men, education status (having no university degree vs at least a baccalaureate degree) was associated with lower sedentary time.

**Table 3:** Correlates of sedentary time (unstandardized beta, 95%CI, p-value)

	Women		Men		Women		Men	
	Model 1: adjusted for multiple sociodemographic variables				Model 2: adjusted for sociodemographics and co-occurring behaviours			
Sociodemographic domain								
Ethnicity (vs not Caucasian)	*Not included*		*Not included*					
Mother’s education (vs no university)	**32.1 (11.3, 52.9)**	**0.002**	*Not included*					
Education (vs no university)	*Not included*		**32.1 (4.8, 59.5)**	**0.021**				
Work/Study Status (vs neither)		0.113		0.**001**				
*Part-time*	−19.4 (−57.9, 19.1)	0.324	−24.3 (−67.6, 19.0)	0.271				
*Full time studying*	14.2 (−19.7, 48.2)	0.412	8.0 (−29.0, 45.1)	0.671				
*Full time working*	7.3 (−27.7, 42.3)	0.682	−45.3 (−81.2, −9.4)	0.013				
Relationship (vs single)		0.189	*Not included*					
*Relationship not living together*	19.0 (−1.8, 39.8)	0.073						
*Relationship living together or married*	11.7 (−12.8, 36.2)	0.349						
Behavioural Domain								
Fruit & Veg (serves/day)	−1.1 (−3.5, 1.3)	0.365	2.6 (−0.8, 6.0)	0.136	*Not included*		2.4 (−1.0, 5.6)	0.169
Energy (kj/kg/day)	−0.1 (−0.3, 0.1)	0.199	−0.1 (− 0.3, 0.04)	0.143	*Not included*		− 0.1 (− 0.3, 0.05)	0.162
Alcohol (drinks/week)	**−1.9 (− 3.1, − 0.6)**	**0.003**	**− 1.0 (− 1.8, − 0.3)**	**0.007**	**−1.4 (−2.7, − 0.1)**	**0.042**	**−1.0 (− 1.7, − 0.2)**	**0.010**
Smoking (vs non-smoking)	**−44.3 (−72.8, − 15.9)**	**0.002**	−15.6 (− 48.1, 16.8)	0.346	**−33.8 (63.8, − 3.9)**	**0.027**	*Not included*	
Sleep Quality (PQSI)	−0.1 (−4.1, 3.8)	0.949	0.6 (−4.0, 5.1)	0.809	*Not included*		*Not included*	
Physical & Psychological Health Domain								
Waist Circumference (cm)	−0.3 (−1.0, 0.3)	0.323	−0.4 (−1.4, 0.7)	0.513	−0.3 (− 1.0, 0.3)	0.314	− 0.7 (− 1.8, 0.5)	0.244
Diagnosed disorders (#)	3.9 (− 0.7, 8.5)	0.100	1.7 (−5.0, 8.3)	0.621	3.5 (−1.1, 8.0)	0.136	2.0 (−4.5, 8.5)	0.553
Physical Health (SF12) (0–100 scale)	0.2 (−1.2, 1.6)	0.780	1.0 (−1.5, 3.5)	0.412	0.2 (−1.1, 1.5)	0.764	1.01 (−1.4, 3.5)	0.420
DASS depression (0–42 scale)	−0.3 (−1.4, 0.8)	0.591	**2.0 (0.4, 3.5)**	**0.014**	−0.2 (−1.2, 0.9)	0.757	**1.9 (0.4, 3.5)**	**0.014**
DASS anxiety (0–42 scale)	−1.2 (−2.8, 0.4)	0.156	0.3 (− 2.1, 2.7)	0.804	−0.6 (− 2.2, 1.1)	0.503	0.6 (− 1.8, 2.9)	0.627
DASS stress (0–42 scale)	−0.6 (− 1.7, 0.5)	0.271	0.6 (− 1.1, 2.2)	0.494	−0.4 (− 1.4, 0.7)	0.466	0.8 (− 0.8, 2.4)	0.341
Vigilance (Cogstate – Identification, log10 reaction time (s))	−50.4 (− 182.3, 81.4)	0.453	−71.7 (− 257.8, 114.3)	0.450	−39.0 (− 167.5, 89.5)	0.551	−67.6 (− 249.6, 114.5)	0.467
Speed of processing (Cogstate – Detection, log10 reaction time (s))	−9.8 (− 105.6, 86.0)	0.841	62.3 (−87.6, 212.2)	0.415	1.0 (− 92.4, 94.4)	0.983	55.8 (−90.7, 202.4)	0.455
Attention & working memory (Cogstate – One Back, log10 reaction time (s))	−20.7 (− 124.5, 83.0)	0.695	−71.7 (− 201.3, 57.9)	0.278	−17.7 (− 118.9, 83.5)	0.732	−66.9 (− 194.1, 60.3)	0.303

Model 1 adjusted for- Women: mother’s education, work/study, relationship status; Men: education, work/study

Model 2 adjusted for- Women: mother’s education, work/study, relationship status, alcohol, smoking; Men: education, work/study, fruit&vegetable servings, energy, alcohol

Bold indicates a statistically significant correlate at p<.05

In the behavioural domain for young women, more alcoholic drinks per week (1.9 min/day, 95%CI: -3.1, − 0.6, *p* = 0.003) and smoking (− 44.3 min/day vs smokers, 95%CI: -72.8, − 15.9, *p* = 0.002) were significantly associated with less sedentary time when adjusted for sociodemographic variables (Table 3, Model 1). The relationships remained significant when further adjusted for other behaviours (Table 3, Model 2). For young men, the only statistically significant correlate of sedentary time was alcoholic drinks consumed; here more alcoholic drinks per week were associated with less sedentary time (− 1.0 min/day, 95%CI: -1.8, − 0.3, *p* = 0.011) when adjusted for sociodemographic variables and remained significant when further adjusted for other behaviours.

In the physical & psychological health domain for young women, no health variables were associated with sedentary time in either model. For young men, lower depression scores were associated with less sedentary time when adjusted for sociodemographic variables (see Table 3 Model 1) and further adjusted for other behaviours (see Table 3 Model 2).

The full models, both for MVPA and sedentary time, restricted to participants with three or more days of valid activity monitor data (*n* = 196 women, *n* = 167 men) provided substantively similar results to the main results, hence, results are presented for the larger sample.

## Discussion

This study examined the correlates of objectively assessed MVPA and sedentary time in young adults across a number of domains. Higher MVPA was associated with a relationship status of single in young women and better sleep quality in young men. Lower sedentary time was associated with having a mother with no university degree, more alcoholic drinks and smoking in young women and not having a university degree, more alcoholic drinks, and lower depression scores in young men.

No prior study to our knowledge has examined multiple domains of correlates for both accelerometer measured MVPA and sedentary time in young adults. Two studies specific to young adults did examine multiple correlates of physical activity [36, 37]. Cleland et al., in a study of young Australian adults, found higher self-reported physical activity was associated with no university education, more alcoholic drinks and being a smoker [36]. They also reported higher daily steps was associated with blue-collar work and higher self-rated health. However, they did not conduct the analyses separated by sex. Dowda et al. examined sex-specific correlates in a study of US young adults ranging from age 18 to 30 and found that in both men and women, higher self-reported MVPA was associated with higher levels of education and not being married [37]. While the current study found higher sedentary time and lower MVPA was associated with higher education, it similarly found being married to be associated with poorer activity levels in women. Health interventions may be able to use marriage as a key life transition period to target behaviours, particularly for women.

The relationships with poor health behaviours such as drinking and smoking with positive activity behaviours (more MVPA; less sedentary time), and the lack of associations with physical and psychological health variables, are different from previous findings in older or mixed-age adult populations which have found associations [2, 3]. Only one previous study to our knowledge has directly compared correlates of sedentary time between age groups [38]. Bernaards et al. found that in young adults, having a sedentary occupation, living in an urban area and being overweight were associated with higher levels of sedentary time whereas in adults 30 years and older higher education status was associated with higher levels of sedentary time [38]. The changes in correlates at different ages highlights the importance of age-specific analysis to account for changes such as in the nature of work participation and family responsibilities.

The magnitude of the effects for sociodemographic correlates was substantial. Women who were living with their partner or married had approximately 30% lower MVPA (approximately 8–10 min/day) compared to single women. Physical activity interventions for young adults may target women living with their partners and through tailored marketing to recruit these women and encourage them to participate in activities involving both women and their partners. Higher education (for women whose mother had a university degree and men with a university degree) also had a substantial effect of approximately 30 min more sedentary time per day, which is of similar magnitude to long-term effects of workplace interventions to decrease sitting time [39]. Higher mother’s education suggests a higher socioeconomic status and potentially more sedentary jobs. While workplace interventions have seen effects in mid to older adult populations with a mean age in their 40s who are likely to have health conditions, these young women also may be key targets for workplace interventions to reduce sedentary time [39].

The magnitude of effects for other health behaviours correlates varied. Poorer sleep quality (the only health behaviour significantly associated with MVPA) had only a small effect size for MVPA in men (1 point higher on 16 point scale associated with 3% lower MVPA, approximately 1 min/day difference). For sedentary time, the effect was substantial for smoking status in women, where being a smoker was associated with 44 min less sedentary time on average. In contrast, although increased alcohol consumption was significantly associated with lower sedentary time for both men and women, the impact was small, with an increase of one alcoholic drink per week associated with only 1–2 min less sedentary time per day. While the individual effects of each correlate may be small, when considered together, they suggest a lifestyle pattern of young adults who are more active and less sedentary but smoke and drink more. In the Australian context this may be those who are more socially active [40]. Interventions need to be aware of the potentially opposing interplay of multiple health behaviours i.e., decreasing smoking may increase sedentary time as well as differential health effects from activity in different contexts. Thus, interventions may need to target these unintended health behaviour changes to achieve a balance of behaviours for best overall health outcomes. In young adults, this may provide physical activity opportunities that are not combined with events where alcohol is consumed and further educating young adults, particularly in the sporting context, how alcohol may negatively affect their sporting performance.

Lastly, there were no strong or statistically significant relationships between any of the physical or psychological health variables and either MVPA or sedentary time in this population, except a small effect of sedentary time on mental health in men. Previous research has suggested 0.5 standard deviations constitutes a minimally important difference for physical activity outcomes (i.e., 27 min of MVPA or 96 min of sedentary time in the current study) [41]. Confidence intervals indicated that non-significant effects were unlikely to be of this magnitude, apart from perhaps the cognitive measures; however, weak effects may have been missed. In contrast to older populations [2, 3], the current study found no strong relationships of MVPA or sedentary time with a count of morbidities, despite high prevalence of some disorders such as spinal pain [42]. It is possible that the detrimental associations between low MVPA and high sedentary time with comorbidities have not had sufficient time to develop at age 22. This may be due to a lack of sufficient cumulative exposure to detrimental activity levels, or the morbidities are not sufficiently advanced in disease progression to limit activity. This may make young adulthood a key period to intervene to prevent future problems, while the relationships between health and activity are still malleable and young adults are still establishing behaviours that track into later adulthood where physical activity, particularly leisure time physical activity, is more consistent [43]. Interventions may be developed together with universities and employers to integrate physical activity and sedentary strategies into their curriculum and employee training.

This study’s strengths included the use of objective measures of activity. A wide variety of correlates across domains were considered, however, some potential correlates were not able to be examined due to low prevalence in the sample (e.g. having children) or lack of data (i.e. potential environmental correlates). Other limitations included the sample size was modest and had a slightly higher socioeconomic status than the whole Raine Study, although there was still a substantial representation of individuals with lower socioeconomic status. Future studies may explore advanced statistical modelling using the distributions and variability found among this young adult population in the current study. The study included a single cohort in one geographical location, thus these findings may not be generalizable to other young adult populations.

## Conclusion

The current results suggest young adults may have unique correlates of MVPA and sedentary time. The relationships of sociodemographics, other health behaviours and physical and psychological health status with activity need to be understood to design effective and efficient interventions at this critical life stage when behaviours are changing and lifetime habits are being established. To optimise health interventions and health outcomes, multiple health behaviours such as alcohol consumption and smoking may need to be accounted for in interventions for young adults.

## Additional file

Additional file 1: **Table S1.** Description of variables included as potential correlates. **Table S2.** Individual correlates (unadjusted except for weartime and number of valid days). **Table S3.** Description of sample comparing included sample with available sample. (DOCX 45 kb)

## Abbreviations

(DASS-21): Depression Anxiety Stress Scales
(MVPA): Moderate-to-vigorous physical activity
