# Supplementary material for: Correlates of physical activity and sedentary time in young adults: the Western Australian Pregnancy Cohort (Raine) Study
Source: BMC Public Health. 2018 Jul 25;18:916. doi: 10.1186/s12889-018-5705-1 (PMC6060463; doi:10.1186/s12889-018-5705-1)
Supplement: Supplementary file 1 — Table S1. Description of variables included as potential correlates. Table S2. Individual correlates (unadjusted except for weartime and number of valid days). Table S3. Description of sample comparing included sample with available sample. (DOCX 45 kb) [file 12889_2018_5705_MOESM1_ESM.docx]

**Supplementary material**

**Supplementary Table A:** Description of variables included as potential correlates

| Domain | Variable | Measure | Type | Reference Justification for inclusion |
| --- | --- | --- | --- | --- |
| Sociodemographic | Sex | Single item | Binary (M/F) | Bauman^5^ (PA), Rhodes^4^ (SED) |
|  | Ethnicity | Single item | Categorical | Bauman^5^ (PA) |
|  | Mother’s Education | Single item | Categorical | Bauman^5^ (PA), Rhodes^4^ (SED) |
|  | Education | Single item | Ordinal (Highest Education Level Attained) | Bauman^5^ (PA), Rhodes^4^ (SED) |
|  | Working/Studying | Single item | Categorical | Trost^2^ (PA) |
|  | Relationship Status | Single item | Categorical (single, relationship, married) | Trost^2^ (PA), Allender^6^ (PA) |
| Behavioural | Diet | FFQ – fruits and vegetables, and energy/kg | Continuous (serves per day, kj/kg) | Trost^2^ (PA) |
|  | Alcohol | Questionnaire | Continuous (drinks per week) | Trost^2^ (PA) |
|  | Smoking | Single item | Binary (yes/no) | Trost^2^ (PA) |
|  | Sleep | Pittsburgh Quality Sleep Index | Continuous (lower scores are better) | Farnsworth^31^ |
| Physical & Psychological Health | Body Composition | Measured waist circumference | Continuous | Bauman^5^ (PA), Rhodes^4^ (SED) |
|  | Diagnosed disorders | # currently diagnosed disorders | Count of binary (yes/no) | Bauman^5^ (PA), Rhodes^4^ (SED) |
|  | Physical Health | SF-12 – physical component | Continuous (higher scores are better) | Bauman^5^ (PA) |
|  | Mental Health | DASS21 depression | Continuous (lower scores are better) | Trost^2^ (PA), Rhodes^4^ (SED) |
|  |  | DASS 21 anxiety | Continuous (lower scores are better) | Trost^2^ (PA), Rhodes^4^ (SED) |
|  |  | DASS21 stress | Continuous (lower scores are better) | Trost^2^ (PA), Rhodes^4^ (SED) |
|  | Cognitive Performance | Cogstate – identification | Continuous (lower scores are better) | Steinmo^32^ (PA) |
|  |  | Cogstate – detection | Continuous (lower scores are better) | Steinmo^32^ (PA) |
|  |  | Cogstate – sets task | Continuous (lower scores are better) | Steinmo^32^ (PA) |

**Supplementary Table B** Individual correlates (unadjusted except for weartime and number of valid days)

|  | MVPA | | | | Sedentary | | | |
| --- | --- | --- | --- | --- | --- | --- | --- | --- |
|  | Women | | Men | | Women |  | Men |  |
|  | RR (95%CI) | p-value | RR (95%CI) | p-value | RR (95%CI) | p-value | RR (95%CI) | p-value |
| **Sociodemographic domain** | | | | | | | | |
| Ethnicity (vs not Caucasian) | 1.20 (.96, 1.51) | 0.108 | 1.01 (.78, 1.30) | 0.960 | -22.8 (-48.8, 3.3) | 0.086 | -2.8 (-36.9, 31.4) | 0.874 |
| Mother’s education (vs no university) | 1.07 (.90, 1.28) | 0.444 | 1.19 (.98, 1.45) | 0.075 | 32.0 (11.8, 52.2) | 0.**002** | 13.9 (-12.2, 40.1) | 0.296 |
| Education (vs no university) | 1.16 (.98, 1.37) | 0.079 | 1.04 (.85, 1.29) | 0.689 | -1.7 (-21.0, 17.6) | 0.862 | 37.0 (9.3, 64.6) | **0.009** |
| Work/Study Status (vs neither) |  | 0.409 |  | 0.469 |  | **0.025** |  | **<0.001** |
| *Part-time* | 1.09 (.77, 1.54) | 0.615 | 1.16 (.83, 1.62) | 0.383 | -25.4 (-64.4, 13.5) | 0.201 | -21.4 (-65.1, 22.3) | 0.337 |
| *Full time studying* | 1.14 (.84, 1.54) | 0.411 | .94 (.71, 1.26) | 0.696 | 15.5 (-19.0, 49.9) | 0.378 | 4.8 (-32.5, 42.1) | 0.801 |
| *Full time working* | .98 (.71, 1.33) | 0.874 | .94 (.71, 1.24) | 0.672 | 0.4 (-35.0, 35.9) | 0.981 | -50.8 (-86.8, -14.8) | **0.006** |
| Relationship (vs single) |  | **0**.**004** |  | 0.876 |  | 0.292 |  | 0.157 |
| *Relationship not living together* | .88 (.74, 1.05) | 0.163 | .98 (.880, 1.19) | 0.807 | 15.4 (-5.5, 36.4) | 0.149 | -25.6 (-52.0, 0.8) | 0.057 |
| *Relationship living together or married* | .70 (.57, .86) | **0**.**001** | .94 (.72, 1.21) | 0.612 | -0.2 (-24.6, 24.2) | 0.989 | -15.6 (-49.8, 18.6) | 0.371 |
| **Behavioural Domain** | | | | | | | | |
| Fruit & Veg (serves/day) | 1.01 (.99, 1.03) | 0.330 | .996 (.97, 1.02) | 0.768 | -0.9 (-3.4, 1.6) | 0.491 | 3.0 (-0.5, 6.5) | 0.092 |
| Energy (kj/kg/day) | 1.00 (.999, 1.002) | 0.580 | 1.001 (.999, 1.002) | 0.150 | -0.1 (-0.2, 0.1) | 0.319 | -0.1 (-0.3, 0.1) | 0.294 |
| Alcohol (drinks/week) | 1.01 (.999, 1.02) | 0.065 | 1.003 (.997, 1.01) | 0.351 | -2.1 (-3.3, -0.9) | **0.001** | -1.3 (-2.0, -0.5) | **0.001** |
| Smoking (vs non-smoking) | 1.13 (.88, 1.46) | 0.338 | .96 (.74, 1.23) | 0.722 | -48.2 (-76.9, -19.4) | **0.001** | -25.8 (-59.4, 7.7) | **0.131** |
| Sleep Quality (PQSI) (0-16 scale) | .98 (.94, 1.01) | 0.204 | .97 (.93, 1.001) | 0.060 | -0.9 (-5.0, 3.1) | 0.654 | 0.1 (-4.6, 4.8) | 0.963 |
| **Physical & Psychological Health Domain** | | | | | | | | |
| Waist Circumference (cm) | .997 (.99, 1.00) | 0.302 | 1.002 (.99, 1.01) | 0.550 | -0.5 (-1.1, 0.1) | 0.131 | -0.5 (-1.6, 0.7) | 0.355 |
| Diagnosed disorders (#) | .96 (.92, 1.00) | 0.063 | .97 (.92, 1.02) | 0.208 | 2.6 (-2.1, 7.3) | 0.278 | 1.5 (-5.3, 8.4) | 0.660 |
| Physical Health (SF12) (0-100 scale) | 1.01 (.999, 1.02) | 0.065 | 1.002 (.98, 1.02) | 0.813 | 0.5 (-0.9, 1.9) | 0.461 | 1.9 (-0.6, 4.4) | 0.140 |
| DASS depression (0-21 scale) | .99 (.98, 1.00) | 0.051 | 1.00 (.99, 1.01) | 0.976 | -0.7 (-1.8, 0.4) | 0.232 | 2.1 (0.4, 3.7) | **0.012** |
| DASS anxiety (0-21 scale) | .996 (.98, 1.01) | 0.594 | .99 (.97, 1.01) | 0.265 | -1.5 (-3.2, 0.2) | 0.075 | 0.5 (-1.9, 3.0) | 0.667 |
| DASS stress (0-21 scale) | .996 (.99, 1.01) | 0.397 | 1.00 (.98, 1.01) | 0.486 | -0.8 (-1.9, 0.3) | 0.162 | 1.0 (-0.7, 2.6) | 0.268 |
| Vigilance (Cogstate – Identification, log10 reaction time in seconds) | 1.15 (.35, 3.72) | 0.822 | 1.01 (.24, 4.25) | 0.992 | -47.2 (-183.5, 89.1) | 0.497 | -127.2 (-320.2, 65.8) | 0.196 |
| Speed of processing (Cogstate – Detection, log10 reaction time in seconds) | 1.20 (.52, 2.78) | 0.676 | .72 (.23, 2.26) | 0.574 | 15.9 (-81.7, 113.5) | 0.750 | 10.4 (-144.1, 165.0) | 0.895 |
| Attention & working memory (Cogstate – One Back, log10 reaction time in seconds) | .80 (.32, 1.99) | 0.631 | 2.12 (.79, 5.71) | 0.137 | -35.0 (-140.7, 70.7) | 0.517 | -93.6 (-227.5, 40.3) | 0.171 |

**Supplementary Table C:** Description of sample comparing included sample with available sample

The available sample size varied for each variable, thus the total available Ns are presented. There were no differences in MVPA, sedentary time, or average wear-time between the included and excluded women. Men in the included sample had a higher average wear-time and average sedentary time per day compared to those with accelerometer data but excluded from the analysed sample. There was a higher percentage of women in the included sample compared to those excluded. In the included sample, a higher percentage of women had mothers with university degrees, completed university, and were single. A higher percentage of men in the included sample had mothers with a university degree. Behavioural and health variables were similar between the included and excluded samples except for the Cogstate measures of vigilance, speed of processing, and attention which were poorer in the excluded sample for both women and men. Additionally, men in the included sample reported fewer alcoholic drinks compared to those who were excluded from the analyses.

|  |  | Included |  |  | Excluded |  | p-value comparing included and excluded* (women, men) |
| --- | --- | --- | --- | --- | --- | --- | --- |
| Domain | Variable | Women (n=256) | Men  (n=219) | N available (women, men) | Women | Men |  |
| Accelerometer Variables | MVPA (average min/ day) | 27.0 (16.2, 41.3) | 34.1 (20.3, 52.1) | 134, 165 | 26.2 (16.2, 38.2) | 35.5 (21.2, 57.2) | 0.625, 0.249 |
|  | Sedentary (average min/day) | 569.7 (85.1) | 549.1 (92.8) | 134, 165 | 554.4 (98.9) | 515.5 (105.1) | 0.112, **0.001** |
|  | Weartime (average min/day) | 903.3 (90.7) | 905.3 (89.3) | 134, 165 | 898.9 (95.9) | 879.1 (100.8) | 0.656, **0.008** |
| Sociodemographic | Ethnicity (% Caucasian mother and father) | 218 (85.2%) | 186 (84.9%) | 1,158, 1,235 | 931 (80.4%) | 1,033 (83.6%) | 0.077, 0.633 |
|  | Mother’s education (university vs no university) | 70 (27.3 %) | 65 (29.7%) | 738, 845 | 122 (16.5%) | 144 (17.0%) | **<0.001, <0.001** |
|  | Education (university vs no university) | 89 (34.8%) | 52 (23.7%) | 337, 289 | 89 (26.4%) | 64 (22.2%) | **0.028**, 0.671 |
|  | Studying/Working Status (vs neither) |  |  | 351, 320 |  |  | **<0.001**, 0.997 |
|  | *Part-time* | 42 (16.4%) | 31 (14.2%) |  | 69 (19.7%) | 46 (14.4%) |  |
|  | *Full time studying* | 112 (43.8%) | 67 (30.6%) |  | 92 (26.2%) | 95 (29.7%) |  |
|  | *Full time working* | 81 (31.6%) | 89 (40.6%) |  | 145 (41.3%) | 131 (40.9%) |  |
|  | Relationship (vs single) |  |  | 335, 281 |  |  | **0.003**, 0.401 |
|  | *Relationship not living together* | 90 (35.2%) | 76 (34.7%) |  | 111 (33.1%) | 82 (29.2%) |  |
|  | *Relationship living together or married* | 54 (21.1%) | 36 (16.4%) |  | 111 (33.1%) | 53 (18.9%) |  |
| Behavioural | Diet |  |  |  |  |  |  |
|  | *Fruit & Vegetable (serves/day)* | 7 (1, 8) | 6 (1, 8) | 369, 362 | 7 (5, 8) | 6 (1, 8) | 0.754, 0.755 |
|  | *Total energy (kJ/kg/day)* | 89.0 (66.5, 116.6) | 112.2 (83.6, 150.2) | 274, 315 | 91.5 (64.4, 116.9) | 110.5 (83.2, 147.0) | 1.00, 0.964 |
|  | Alcohol (drinks/wk) | 3.1 (0.6, 7.5) | 6 (1.3, 14.5) | 341, 298 | 2.5 (0.4, 6.8) | 9.6 (2.4, 18.0) | 0.222, **0**.**011** |
|  | Smoking | 31 (12.1%) | 35 (16.0%) | 347, 317 | 54 (15.6%) | 67 (21.1%) | 0.228, 0.135 |
|  | Sleep quality (0-16, higher scores indicate poorer sleep) | 4 (3, 6) | 4 (3, 6) | 297, 251 | 5 (3, 6) | 4 (3, 5) | 0.143, 0.199 |
| Physical & Psychological Health | Waist Circumference (cm) | 80.0 (14.0) | 85.2 (11.2) | 274, 318 | 81.5 (16.4) | 86.6 (12.4) | 0.251, 0.192 |
|  | # Diagnosed disorders (current) | 2 (1, 3) | 1 (0, 2) | 364, 337 | 2 (1, 3) | 1 (0, 2) | 0.816, 0.532 |
|  | SF-12 physical component | 53.5 (6.6) | 54.8 (4.8) | 322, 265 | 52.9 (7.6) | 54.7 (5.5) | 0.351, 0.737 |
|  | DASS-21 (score range 0 to 42, higher scores indicate poorer mental health) |  |  |  |  |  |  |
|  | *Depression* | 4 (2, 12) | 2 (0, 8) | 333, 281 | 4 (2, 12) | 2 (0, 8) | 0.756, 0.956 |
|  | *Anxiety* | 4 (0, 8) | 2 (0, 6) | 332, 279 | 2 (0, 8) | 2 (0, 6) | 0.735, 0.864 |
|  | *Stress* | 10 (4, 16) | 6 (2, 10) | 331, 279 | 10 (4, 16) | 6 (2, 10) | 0.604, 0.459 |
|  | Vigilance (Cogstate – Identification, log10 reaction time in seconds) | 2.6 (2.6, 2.7) | 2.6 (2.6, 2.7) | 165, 194 | 2.7 (2.6, 2.7) | 2.6 (2.6, 2.7) | 0.078, **0.028** |
|  | Speed of processing (Cogstate – Detection, log10 reaction time in seconds) | 2.4 (2.4, 2.5) | 2.4 (2.4, 2.5) | 157, 181 | 2.5 (2.4, 2.5) | 2.4 (2.4, 2.5) | 0.075, **0.035** |
|  | Attention & working memory (Cogstate – One Back, log10 reaction time in seconds) | 2.8 (2.8, 2.9) | 2.8 (2.7, 2.8) | 167, 193 | 2.8 (2.8, 2.9) | 2.8 (2.7, 2.9) | **0.018**, 0.076 |

*Continuous data compared by t test, non-normal continuous data compared using Mann Whitney U, categorical data compared with chi-squared
